# Prognostic factors for cardiovascular-related mortality in pediatric hypertrophic cardiomyopathy: a retrospective cohort study with survival analysis and X-tile stratification

**DOI:** 10.3389/fmed.2026.1825591

**Published:** 2026-04-21

**Authors:** Shiguang Li, Ning Zhang, Danyan Su, Cheng Chen, Suyuan Qin, Bingbing Ye, Yusheng Pang

**Affiliations:** 1 Department of Pediatrics, Hainan General Hospital, Hainan Affiliated Hospital of Hainan Medical University, Haikou, China; 2 Department of Pediatrics, The First Affiliated Hospital of Guangxi Medical University, Nanning, Guangxi, China

**Keywords:** cardiovascular-related mortality, Cox regression, Kaplan–Meier analysis, pediatric hypertrophic cardiomyopathy, risk stratification, X-tile

## Abstract

**Objective:**

To identify prognostic factors associated with cardiovascular-related mortality in children with hypertrophic cardiomyopathy (HCM) and to explore outcome-oriented risk stratification using optimal cut-off values for continuous variables.

**Methods:**

This retrospective cohort study included 41 children diagnosed with HCM at The First Affiliated Hospital of Guangxi Medical University between January 1, 2013, and October 1, 2024. Baseline demographic characteristics, clinical manifestations, chest radiographic findings, electrocardiographic features, echocardiographic parameters, and serum biochemical indices were collected. Cardiovascular-related mortality was defined as the primary endpoint, with follow-up censored at the last clinical contact for patients lost to follow-up. Univariate Cox proportional hazards regression was performed to screen variables associated with mortality. Optimal cut-off values for continuous variables were determined using X-tile software, followed by Kaplan–Meier survival analysis and log-rank testing to compare survival differences between risk strata.

**Results:**

Univariate Cox analysis showed no significant associations between baseline categorical clinical variables and cardiovascular-related mortality, with only a borderline sex-related difference observed. In contrast, several echocardiographic parameters were significantly associated with mortality. Increased left ventricular end-diastolic diameter (LVEDd) (HR = 1.084, *p* = 0.049), left ventricular end-systolic diameter (LVESd) (HR = 1.136, *p* = 0.018), interventricular septal thickness (IVSd) (HR = 1.145, *p* = 0.037), and left ventricular posterior wall thickness (LVPWd) (HR = 1.718, *p* = 0.001) were associated with higher risk, whereas higher left ventricular ejection fraction (LVEF) (HR = 0.953, *p* = 0.020) and left ventricular fractional shortening (LVFS) (HR = 0.924, *p* = 0.016) were associated with reduced risk. Elevated creatine kinase (CK), lactate dehydrogenase (LDH), cardiac troponin I (cTnI), aspartate aminotransferase (AST), and alanine aminotransferase (ALT) were also significantly associated with increased mortality (all *p* < 0.05). X-tile–derived cut-off values further stratified survival, with significantly worse outcomes observed in patients above threshold values for LVEDd (38 mm), LVESd (23 mm), IVSd (12 mm), LVPWd (9 mm), CK (179 U/L), LDH (416 U/L), cTnI (0.06 ng/mL), AST (44 U/L), and ALT (38 U/L), and below threshold values for LVEF (60%) and LVFS (32%) (multiple *p* < 0.05, several *p* < 0.001).

**Conclusion:**

This single-center real-world cohort suggests that cardiovascular-related mortality in pediatric HCM is more consistently associated with echocardiographic indicators reflecting left ventricular remodeling, hypertrophic burden, and systolic function, as well as biochemical markers of myocardial injury and metabolic stress. X-tile–based stratification of continuous variables provides intuitive and potentially actionable risk thresholds for intra-cohort follow-up and early risk warning. However, the generalizability of these thresholds requires validation in larger, multicenter cohorts.

## 1 Introduction

### 1.1 Background related to pediatric hypertrophic cardiomyopathy

Pediatric hypertrophic cardiomyopathy (HCM) is one of the most common inherited cardiomyopathies in childhood and is characterized by myocardial hypertrophy, reduced left ventricular compliance, impaired cardiac function, and a wide spectrum of arrhythmic manifestations (1–3). Among children and adolescents, HCM represents a major cause of sudden cardiac death of cardiac origin (4). Compared with adult HCM, pediatric HCM exhibits greater heterogeneity in age at onset, etiological composition, clinical phenotype, and disease trajectory. Some patients experience rapid disease progression during infancy or early childhood, presenting with overt heart failure, whereas others develop nonspecific symptoms such as chest pain, syncope, or reduced exercise tolerance during school age or adolescence, and may even present with severe adverse events as the initial manifestation (5–7). The etiological spectrum of pediatric HCM is also more complex. In addition to sarcomeric protein–encoding gene mutations, HCM in children may be associated with metabolic disorders, mitochondrial diseases, or syndromic conditions (2, 8, 9). This marked heterogeneity poses a major challenge for early identification of high-risk pediatric patients and for the development of prognostic assessment tools that are both interpretable and clinically actionable, which remains a key unmet need in the clinical management and research of pediatric HCM.

Previous studies have suggested that adverse outcomes in pediatric HCM may be associated with multiple factors, including discrete clinical characteristics (such as age at diagnosis, sex, family history, and presenting symptoms), cardiac structural and functional parameters, electrocardiographic abnormalities, and selected biomarkers related to myocardial injury or stress. However, owing to small sample sizes, low event rates, heterogeneous etiological composition, and variability in follow-up strategies, existing findings have been inconsistent. Further validation based on real-world cohorts is therefore required to identify stable predictors of mortality and to support risk stratification during follow-up (10–14).

### 1.2 Significance of the combined application of Cox regression, X-tile, and Kaplan–Meier survival analysis

Cox proportional hazards regression is widely used in prognostic research to evaluate associations between candidate variables and time-to-event outcomes, providing hazard ratios and confidence intervals and enabling systematic screening of potential risk factors for mortality (15–17). In pediatric HCM, many clinically relevant indicators—such as ventricular dimensions, wall thickness, systolic function, and biochemical markers—are continuous variables. Reliance on empirically defined cut-off values may therefore limit sensitivity and comparability across studies. X-tile software enables data-driven identification of optimal cut-off values under a survival outcome framework, allowing continuous variables to be categorized in a manner aligned with cohort-specific characteristics and enhancing risk discrimination in Kaplan–Meier survival analysis (18). Kaplan–Meier curves, together with log-rank testing, provide an intuitive visualization of survival differences across risk strata and offer complementary validation of associations identified through Cox regression.

Accordingly, the present study adopted a sequential analytical strategy combining univariate Cox regression, X-tile–based determination of optimal cut-off values for continuous variables, and Kaplan–Meier survival analysis. Using a real-world cohort of 41 children with HCM, we systematically evaluated the associations between clinical characteristics, echocardiographic parameters, serum biochemical indices, and cardiovascular-related mortality, with the aim of providing more actionable evidence to support early risk identification and individualized follow-up management in pediatric HCM.

## 2 Materials and methods

### 2.1 Study design and study population

This retrospective single-center cohort study included children diagnosed with hypertrophic cardiomyopathy (HCM) at The First Affiliated Hospital of Guangxi Medical University between January 1, 2013, and October 1, 2024. The study population was derived from an established pediatric HCM database previously reported by our research group, with partial overlap in study center, enrollment period, and follow-up framework (19). A total of 41 patients were included in the present analysis.

Clinical data collection was consistent with the previously reported cohort and included demographic characteristics, clinical manifestations, laboratory findings, electrocardiography, chest radiography, transthoracic echocardiography, treatment information, and follow-up data. In contrast to the prior study, which primarily focused on clinical characteristics, etiological classification, and genetic findings, the present study is specifically designed to investigate prognostic factors associated with cardiovascular-related mortality. Furthermore, the current study adopts a survival-oriented analytical framework, incorporating univariate Cox proportional hazards regression and X-tile–based cut-off optimization to identify risk-associated variables and establish outcome-driven risk stratification. These analyses were not included in the previous publication.

### 2.2 Inclusion and exclusion criteria

Patients were eligible for inclusion if they met the following criteria: (1) age <18 years at initial diagnosis; (2) a diagnosis of HCM established based on integrated assessment of clinical features, imaging findings (primarily echocardiography), genetic testing, and metabolic screening when indicated; and (3) fulfillment of guideline-recommended diagnostic criteria for pediatric HCM, defined as left ventricular wall thickness exceeding age-, sex-, and body surface area–adjusted reference values by more than two standard deviations (*Z*-score > 2) (20). (4) Etiological stratification was performed as follows: myocardial hypertrophy attributable to pathogenic or likely pathogenic variants in sarcomeric protein–encoding genes was classified as primary HCM, whereas hypertrophy associated with inherited metabolic disorders, syndromic conditions, or neuromuscular diseases was classified as secondary HCM. Patients with undetermined or unclassified etiology were analyzed as a separate subgroup, using the same stratification framework as that applied in the previously reported cohort (2).

Exclusion criteria included: (1) Left ventricular hypertrophy attributable to increased afterload conditions (such as hypertension, aortic stenosis, or coarctation of the aorta); (2) physiological hypertrophy related to athletic training or other clearly non-HCM hypertrophic phenotypes; (3) transient myocardial hypertrophy in neonates or infants born to mothers with diabetes; (4) myocardial hypertrophy associated with long-term corticosteroid use or other medications; and (5) extensive missing key clinical data precluding reliable assessment of major variables or determination of outcome events.

### 2.3 Data collection and variable definitions

Baseline clinical data included sex, age at diagnosis, family history, presenting symptoms, cardiac murmurs, and extracardiac features. Heart failure status was assessed using the modified Ross classification in children younger than 3 years and the New York Heart Association (NYHA) functional classification in older patients. Chest radiographs were reviewed for cardiomegaly, and standard 12-lead electrocardiograms were evaluated for ST-T changes, pathological Q waves, QT interval prolongation, ventricular hypertrophy, and conduction abnormalities.

Transthoracic echocardiographic parameters included left ventricular end-diastolic diameter (LVEDd), left ventricular end-systolic diameter (LVESd), interventricular septal thickness (IVSd), left ventricular posterior wall thickness (LVPWd), left ventricular ejection fraction (LVEF), left ventricular fractional shortening (LVFS), and selected right-sided measurements. All echocardiographic measurements were independently obtained by two experienced sonographers, and mean values were used for analysis.

Serum biochemical indices included creatine kinase (CK), lactate dehydrogenase (LDH), cardiac troponin I (cTnI, ng/mL), aspartate aminotransferase (AST), alanine aminotransferase (ALT), and renal function markers. Left ventricular outflow tract obstruction was defined based on echocardiographic assessment of the left ventricular outflow tract pressure gradient and used to categorize patients as having obstructive or non-obstructive physiology (21).

Right ventricular outflow tract obstruction was included as an independent binary variable (present/absent) in the analysis.

### 2.4 Endpoint definition and follow-up

Follow-up data were obtained through outpatient visits and telephone interviews and were censored on October 1, 2024. The primary endpoint was cardiovascular-related mortality. Patients lost to follow-up were treated as right-censored at the time of their last confirmed clinical contact (19).

### 2.5 Statistical analysis

Statistical analyses were performed using SPSS software (version 26.0). Univariate Cox proportional hazards regression was used to evaluate associations between candidate variables and cardiovascular-related mortality, with hazard ratios and 95% confidence intervals reported. Variables with *p* < 0.05 were considered potentially prognostic. Kaplan–Meier survival curves and log-rank tests were used to compare survival between groups. For significant continuous variables, optimal cut-off values were determined using X-tile software, followed by Kaplan–Meier analysis. Analyses were conducted based on available data without imputation. Some variables contained missing values. Outliers attributable to measurement error were excluded, whereas extreme values of uncertain origin were retained and assessed through sensitivity analyses.

### 2.6 Ethics statement

This study was approved by the Ethics Committee of Hainan Provincial People’s Hospital (approval no. 2022(404); Haikou, China) and by the Ethical Review Committee of the First Affiliated Hospital of Guangxi Medical University (approval no. 2026-E0113; Nanning, China). All procedures involving human participants were conducted in accordance with the ethical standards of the institutional research committees and with the 2013 revision of the Declaration of Helsinki. Given the retrospective design and the use of de-identified data, the requirement for written informed consent was waived by both committees.

## 3 Results

### 3.1 Baseline characteristics and follow-up overview (Table 1)

**Table 1:** Baseline characteristics and follow-up profile of the study cohort (*N* = 41).

Variable	Overall
Demographic characteristics	
Sex, *n* (%)	
Male	27 (65.9)
Female	14 (34.1)
Age at initial diagnosis, median (range)	4 years 3 months (12 days–14 years 3 months)
Age at diagnosis, *n* (%)	
<1 year	19 (46.3)
1–3 years	10 (24.4)
>3–6 years	4 (9.8)
>6 years	8 (19.5)
Etiology and genetics	
Underwent genetic testing, *n* (%)	24 (58.5)
Etiological classification, *n* (%)	
Primary HCM	13 (31.7)
Secondary HCM	11 (26.8)
Unclassified/undetermined	17 (41.5)
Secondary etiology, *n*	
Noonan syndrome	8
Mitochondrial disease	2
Metabolic disorders	1
Family history of cardiovascular disease, *n* (%)	7 (17.1)
Selected echocardiographic features	
Asymmetric septal hypertrophy, *n* (%)	25 (61.0)
Left ventricular outflow tract obstruction (LVOTO), n (%)	9 (22.0)
Follow-up and outcomes	
End of follow-up	October 1, 2024
Cardiovascular-related death, *n* (%)	7 (17.1)
Lost to follow-up (right-censored), *n* (%)	5 (12.2)
Follow-up duration, median (IQR), months	60.4 (35.9–89.3)
Follow-up duration, range, months	0.4–182.6

Data are presented as *n* (%) for categorical variables and as median (range) or median (interquartile range, IQR) for continuous variables, as appropriate. Patients lost to follow-up were treated as right-censored at the time of last contact. HCM, hypertrophic cardiomyopathy; LVOTO, left ventricular outflow tract obstruction; IQR, interquartile range.

A total of 41 children with hypertrophic cardiomyopathy were included, comprising 27 boys (65.9%) and 14 girls (34.1%). Follow-up was censored on October 1, 2024. During follow-up, 7 patients (17.1%) experienced cardiovascular-related death and 5 patients (12.2%) were lost to follow-up; these patients were treated as right-censored at their last confirmed contact. The median follow-up duration was 60.4 months (IQR, 35.9–89.3 months; range, 0.4–182.6 months).

### 3.2 Univariate Cox regression analysis of clinical characteristics and electrocardiographic variables (Table 2)

**Table 2:** Univariate Cox proportional hazards analysis of clinical characteristics and electrocardiographic findings.

Variable	HR (95% CI)	*p*-value
Age > 1 year vs. ≤ 1 year	2.099 (0.233–18.878)	0.508
Age > 3 years vs. ≤ 3 years	1.730 (0.384–7.792)	0.475
Female sex (vs. Male)	0.149 (0.017–1.304)	0.085
Family history of HCM (no vs. yes)	0.421 (0.077–2.312)	0.319
NYHA functional class II–IV (vs. I)	1.882 (0.416–8.525)	0.412
Respiratory infection at initial diagnosis (yes vs. no)	0.918 (0.203–4.144)	0.911
Recurrent respiratory infections (yes vs. no)	0.505 (0.058–4.400)	0.536
Heart failure at presentation (yes vs. no)	1.616 (0.358–7.282)	0.532
Growth retardation (yes vs. no)	1.296 (0.288–5.820)	0.735
Dyspnea (yes vs. no)	1.237 (0.239–6.405)	0.800
Chest pain or chest tightness (yes vs. no)	1.184 (0.229–6.123)	0.840
Fatigue or reduced exercise tolerance (yes vs. no)	1.666 (0.369–7.523)	0.507
Dysmorphic features or hypotonia (yes vs. no)	0.721 (0.086–6.023)	0.763
Cardiac murmur (yes vs. no)	0.948 (0.183–4.898)	0.949
Cardiomegaly on chest radiograph (yes vs. no)	33.815 (0.015–74669.886)	0.370
ST-T changes on ECG (yes vs. no)	2.330 (0.270–20.370)	0.443
Pathological Q waves (yes vs. no)	1.021 (0.195–5.348)	0.980
Prolonged QT interval (yes vs. no)	0.307 (0.036–2.607)	0.279
Electrocardiographic ventricular hypertrophy (yes vs. no)	1.299 (0.250–6.752)	0.756

HR indicates hazard ratio; CI, confidence interval; HCM, hypertrophic cardiomyopathy; NYHA, New York Heart Association; ECG, electrocardiogram.

In univariate Cox regression analyses, none of the assessed clinical characteristics, chest radiographic findings, or electrocardiographic variables were significantly associated with cardiovascular-related mortality (all *p* > 0.05). Female sex showed a borderline association (HR = 0.149, 95% CI 0.017–1.304; *p* = 0.085).

### 3.3 Univariate Cox regression analysis of echocardiographic and biochemical variables (Table 3)

**Table 3:** Univariate Cox proportional hazards analysis of echocardiographic parameters and biochemical markers.

Variable	HR (95% CI)	*p*-value
Echocardiographic parameters		
Left atrial diameter (LA)	1.032 (0.953–1.118)	0.438
Left ventricular end-diastolic diameter (LVEDd)	1.084 (1.000–1.174)	0.049
Left ventricular end-systolic diameter (LVESd)	1.136 (1.022–1.262)	0.018
Interventricular septal thickness (diastole) (IVSd)	1.145 (1.008–1.300)	0.037
Left ventricular posterior wall thickness (diastole) (LVPWd)	1.718 (1.235–2.390)	0.001
Right ventricular end-diastolic diameter (RV)	1.098 (0.885–1.362)	0.393
Right ventricular outflow tract diameter (RVOT)	1.081 (0.935–1.250)	0.294
Left ventricular ejection fraction (LVEF)	0.953 (0.915–0.993)	0.020
Left ventricular fractional shortening (LVFS)	0.924 (0.867–0.985)	0.016
Atrial enlargement (no vs. yes)	2.512 (0.484–13.039)	0.273
E/A ratio > 1 (vs. ≤ 1)	1.087 (0.171–6.916)	0.930
Biochemical markers		
Creatine kinase (CK)	1.007 (1.002–1.013)	0.012
Creatine kinase-MB (CK-MB)	1.047 (0.995–1.102)	0.074
Lactate dehydrogenase (LDH)	1.006 (1.002–1.011)	0.006
Cardiac troponin I (cTnI)	385923581.7 (331.347–4.495E+14)	0.006
Blood urea nitrogen (BUN)	0.994 (0.903–1.093)	0.894
Creatinine	1.057 (0.997–1.121)	0.063
Creatinine clearance	0.973 (0.936–1.012)	0.173
Gamma-glutamyltransferase (GGT)	1.028 (0.965–1.095)	0.396
Aspartate aminotransferase (AST)	1.026 (1.005–1.047)	0.017
Alanine aminotransferase (ALT)	1.020 (1.004–1.037)	0.017
RVOT obstruction (yes vs. no)	0.024 (0–17.007)	0.266

HR indicates hazard ratio; CI, confidence interval. Echocardiographic linear measurements are reported as diameters (in millimeters), and LVEF and LVFS are reported as percentages.

LA, left atrial diameter; LVEDd, left ventricular end-diastolic diameter; LVESd, left ventricular end-systolic diameter; IVSd, interventricular septal thickness (diastole); LVPWd, left ventricular posterior wall thickness (diastole); RV, right ventricular end-diastolic diameter; RVOT, right ventricular outflow tract diameter; LVEF, left ventricular ejection fraction; LVFS, left ventricular fractional shortening; CK, creatine kinase; CK-MB, creatine kinase-MB; LDH, lactate dehydrogenase; cTnI, cardiac troponin I; BUN, blood urea nitrogen; GGT, gamma-glutamyltransferase; AST, aspartate aminotransferase; ALT, alanine aminotransferase.

Several echocardiographic parameters were significantly associated with cardiovascular-related mortality. Increased LVEDd, LVESd, IVSd, and LVPWd were associated with higher mortality risk, whereas reduced LVEF and LVFS were associated with increased risk.

Among biochemical markers, higher levels of CK, LDH, cTnI, AST, and ALT were associated with increased mortality risk, while other biochemical indices were not significant. The relatively large hazard ratio for cTnI may be related to its measurement scale, as values are expressed in small units (ng/mL), and the effect size reflects the risk per unit increase.

### 3.4 Kaplan–Meier survival analysis: stratification strategies for categorical and continuous variables

To avoid potential interpretative inconsistency arising from mixing categorical and continuous variables within the same analysis, Kaplan–Meier survival analyses were conducted and reported separately according to variable type.

#### 3.4.1 Kaplan–Meier analysis of categorical variables based on original groupings

Kaplan–Meier survival analyses of categorical variables using original groupings showed no significant survival differences across most strata (all log-rank *p* > 0.05). Sex showed a borderline difference in survival (*p* = 0.052), and cardiomegaly on chest radiography was not significantly associated with survival (*p* = 0.135).

#### 3.4.2 Kaplan–Meier analysis of continuous variables stratified by X-tile–derived cut-off values

**Figure 1:**
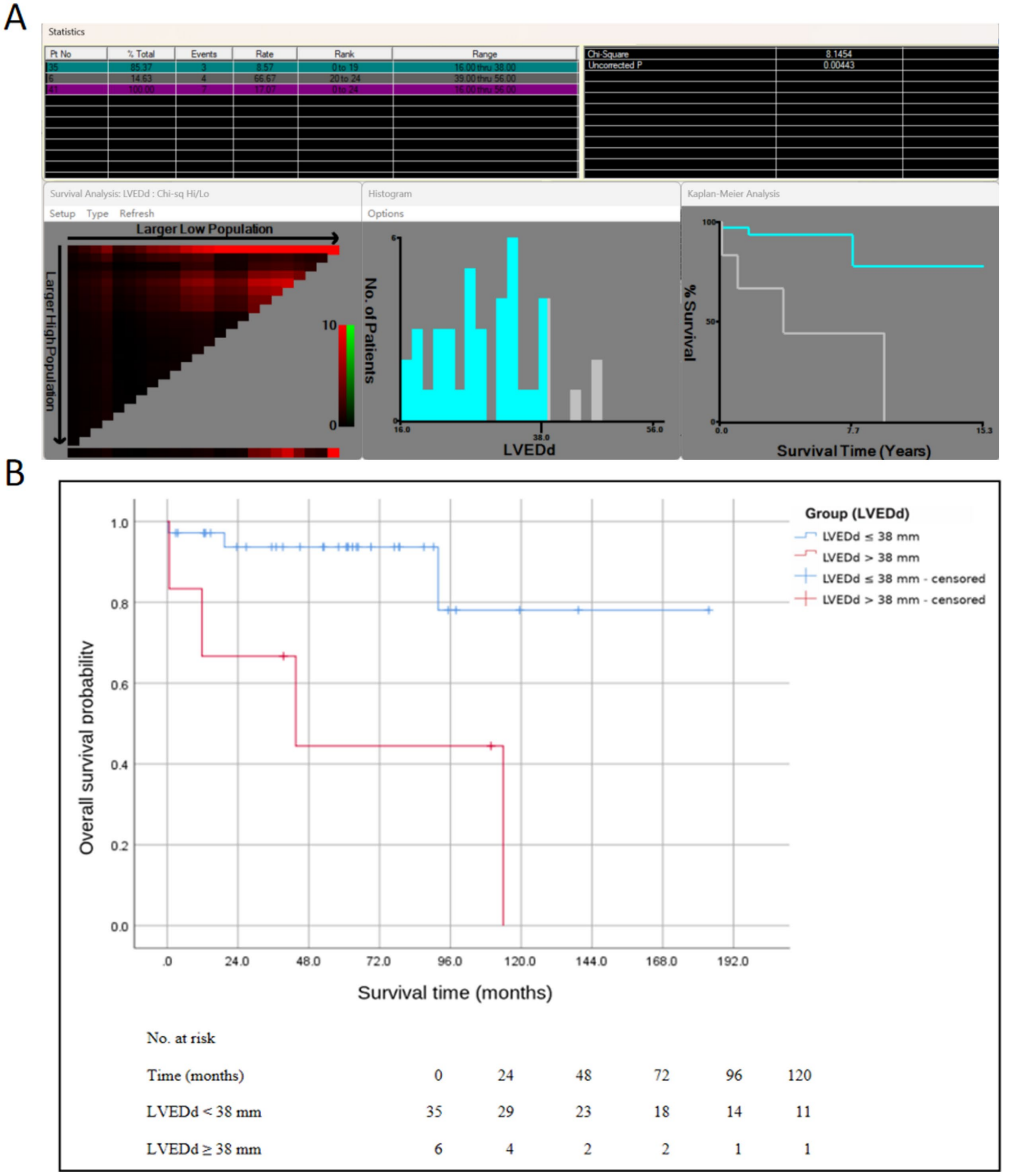
Kaplan–Meier survival curves stratified by LVEDd based on X-tile–derived cut-off values. Patients were divided into groups according to the optimal threshold, and survival differences between groups were compared using the log-rank test. **(A)** X-tile analysis identified 38 mm as the optimal cut-off value for left ventricular end-diastolic diameter (LVEDd). **(B)** Kaplan–Meier survival curves comparing patients with high versus low LVEDd based on the X-tile–derived cut-off value (log-rank test, *p* = 0.004).

For continuous variables that were significant in univariate Cox regression, X-tile–derived cut-off values were applied. Kaplan–Meier analyses based on these cut-offs demonstrated significant survival differences between risk groups (Table 4). Figure 1 presents LVEDd as a representative example.

**Table 4:** Kaplan–Meier survival analysis of continuous variables stratified by X-tile–derived cut-off values.

Variable	X-tile cut-off value	Direction of survival difference	Log-rank *p*-value
LVEDd	38 mm	Worse survival in high-value group	0.004
LVESd	23 mm	Worse survival in high-value group	0.015
IVSd	12 mm	Worse survival in high-value group	0.019
LVPWd	9 mm	Worse survival in high-value group	<0.001
LVEF	60%	Worse survival in low-value group	<0.001
LVFS	32%	Worse survival in low-value group	<0.001
CK	179 U/L	Worse survival in high-value group	<0.001
LDH	416 U/L	Worse survival in high-value group	<0.001
cTnI	0.06 ng/mL	Worse survival in high-value group	0.001
AST	44 U/L	Worse survival in high-value group	0.003
ALT	38 U/L	Worse survival in high-value group	0.032

Cut-off values were determined using X-tile software. Survival differences between groups were assessed using the Kaplan–Meier method and compared by the log-rank test.

LVEDd, left ventricular end-diastolic diameter; LVESd, left ventricular end-systolic diameter; IVSd, interventricular septal thickness (diastole); LVPWd, left ventricular posterior wall thickness (diastole); LVEF, left ventricular ejection fraction; LVFS, left ventricular fractional shortening; CK, creatine kinase; LDH, lactate dehydrogenase; cTnI, cardiac troponin I; AST, aspartate aminotransferase; ALT, alanine aminotransferase.

## 4 Discussion

In this single-center real-world cohort of 41 children with hypertrophic cardiomyopathy (HCM), cardiovascular-related mortality was selected as the study endpoint, with follow-up extending to October 1, 2024. Using univariate Cox regression analysis in combination with X-tile–based survival stratification, the present study identified a coherent pattern of prognostic associations. Adverse outcomes were consistently related to left ventricular structural remodeling and hypertrophic burden (LVEDd, LVESd, IVSd, and LVPWd), impaired systolic function (LVEF and LVFS), and selected biochemical markers reflecting myocardial injury or systemic stress (cTnI, CK, LDH, AST, and ALT). X-tile–derived cut-off values further enabled outcome-oriented risk stratification within the cohort. In contrast, most categorical clinical characteristics and routinely assessed electrocardiographic variables did not demonstrate stable risk discrimination. Given the limited number of outcome events and the univariate nature of the analyses, these findings should be interpreted as exploratory and require validation in larger, independent pediatric HCM cohorts. In comparison with prior pediatric cohorts, the overall direction of our findings aligns with the concept that outcomes in childhood HCM are primarily driven by etiological substrate and the severity of cardiac remodeling and functional impairment, rather than by isolated categorical descriptors. Large pediatric studies have highlighted the prognostic relevance of age at presentation (particularly infancy), underlying etiology (including metabolic or syndromic disease), and baseline functional status (22, 23). Within this framework, our consistent signals across structural remodeling, hypertrophic burden, systolic dysfunction, and injury/stress-related biomarkers may represent a convergent “disease progression axis” that can be pragmatically monitored during follow-up in real-world pediatric practice.

In our cohort, enlargement of left ventricular end-diastolic and end-systolic diameters (LVEDd and LVESd) was consistently associated with cardiovascular-related mortality and demonstrated clear survival separation using X-tile–derived cut-off values. These findings suggest that progressive left ventricular chamber enlargement represents an important phenotypic marker of disease progression in pediatric HCM, potentially reflecting a transition away from a compensated hypertrophic state. Previous studies have demonstrated that left ventricular dilation in HCM is frequently accompanied by increased myocardial fibrosis, reduced ventricular compliance, and a higher risk of chronic heart failure, thereby contributing to adverse long-term outcomes (24–26). In pediatric and adolescent populations, heterogeneous patterns of left ventricular remodeling have been described, in which reductions in maximal wall thickness may occur in parallel with declining systolic function, indicating a potential evolution toward a hypocontractile and decompensated disease stage (27). Moreover, myocardial fibrosis has been shown to impair both atrial and ventricular function and may further accelerate clinical deterioration through adverse electromechanical coupling (28, 29). Taken together, our findings support the concept that left ventricular chamber enlargement in children with HCM reflects maladaptive remodeling rather than benign growth-related variation, underscoring the need for intensified longitudinal surveillance and early reassessment of risk status in this subgroup.

Beyond chamber dimensions, indices of hypertrophic burden—specifically interventricular septal thickness (IVSd) and left ventricular posterior wall thickness (LVPWd)—were also significantly associated with mortality risk in our analysis. Myocardial wall thickness reflects the magnitude of hypertrophy and may be linked to increased myocardial workload, microvascular ischemia, and downstream fibrotic remodeling, all of which contribute to disease progression.

In pediatric HCM, increased wall thickness is frequently associated with underlying genetic abnormalities and may indicate a more severe or earlier-onset disease course. Prior studies have suggested that greater hypertrophic burden is associated with higher risks of severe outcomes, including sudden cardiac death–related events, although pediatric-specific risk profiles differ from those observed in adult populations (30, 31). In particular, thickening of the left ventricular posterior wall may not only reflect elevated ventricular load but also serve as a surrogate marker of diffuse myocardial fibrosis. Within this context, our findings suggest that IVSd and LVPWd capture both the structural severity of hypertrophy and the cumulative etiological burden, reinforcing the value of serial structural assessment rather than reliance on single time-point measurements in pediatric HCM.

The present study demonstrated that reduced left ventricular ejection fraction (LVEF) and left ventricular fractional shortening (LVFS) were associated with adverse outcomes, consistent with previous reports (32). When LVEF declines below 50%, myocardial fibrosis burden and myocardial stiffness have been shown to increase substantially, underscoring the importance of cardiac functional assessment in prognostic management. In several studies involving patients with HCM, the chronic progression toward heart failure has been accompanied by gradual declines in LVEF and LVFS, which may increase the risk of further functional deterioration and adverse outcomes. These observations indicate that impaired systolic function is not merely a reflection of clinical symptoms, but also serves as an early warning signal of long-term unfavorable prognosis (33).

Serum biochemical markers can, to some extent, reflect the burden of tissue injury and metabolic stress. In the present study, elevated creatine kinase (CK; HR = 1.007, 95% CI: 1.002–1.013, *p* = 0.012) and lactate dehydrogenase (LDH; HR = 1.006, 95% CI: 1.002–1.011, *p* = 0.006) were associated with adverse outcomes, suggesting that these markers may serve as readily available indicators of overall disease burden or a decompensated physiological state. It should be emphasized, however, that both CK and LDH are non–cardiac-specific biomarkers. Their circulating levels can be influenced by skeletal muscle injury, hemolysis, and hepatic or systemic tissue hypoxia. Accordingly, their prognostic relevance in this context is more likely to reflect a composite manifestation of systemic stress or decompensation, rather than directly indicating structural myocardial injury. Therefore, the associations of these biomarkers with mortality should be interpreted with caution.

At the myocardial pathological level, previous studies have consistently recognized myocardial fibrosis as a key pathological substrate underlying disease progression and adverse outcomes in HCM, and as being closely associated with the risks of heart failure and arrhythmias (25, 34, 35). In this context, more myocardium-oriented injury markers, such as cardiac troponins, may better reflect ongoing cardiomyocyte injury, adverse cardiac remodeling, and an increased fibrotic burden. Prior evidence has shown that elevations in myocardial injury–related biomarkers, including troponins, are significantly associated with higher risks of all-cause mortality, cardiovascular death, and sudden cardiac death (10). Moreover, increased levels of high-sensitivity cardiac troponin T (hs-cTnT) have been closely linked to adverse cardiac remodeling and a greater extent of myocardial fibrosis (11, 25, 36). In addition, in certain genotype-positive HCM populations, myocardial fibrosis and abnormalities in myocardial energy metabolism may jointly contribute to persistently elevated troponin levels, suggesting a complex pathophysiological mechanism underlying sustained myocardial injury signals (37).

In the univariate Cox regression analysis of the present study, elevated cardiac troponin I (cTnI) was associated with an increased risk of cardiovascular-related mortality (*p* = 0.006; HR = 385,923,581.7). It should be noted that the magnitude of the hazard ratio for cTnI is highly influenced by the unit of measurement and the distribution of values; therefore, emphasis in this study is placed primarily on the direction of the association rather than on the absolute HR estimate. Taken together, elevated cTnI levels in this cohort are more likely to reflect ongoing myocardial injury and adverse remodeling processes, indicating a higher risk of unfavorable outcomes. These findings suggest that cTnI may serve as a useful adjunct in prognostic assessment for pediatric HCM. Nevertheless, the establishment of clinically meaningful thresholds and the generalizability of these results require further validation in larger, multicenter studies.

Aspartate aminotransferase (AST) and alanine aminotransferase (ALT) were also associated with adverse outcomes in the present study, suggesting that these markers may reflect systemic metabolic or hepatic stress under decompensated conditions. Chronic heart failure or states of reduced perfusion can lead to hepatic congestion and elevations in transaminase levels, which often coexist with increased fibrotic burden, heightened ventricular stiffness, and reduced chamber compliance. Accordingly, AST and ALT are more appropriately interpreted as auxiliary risk signals, and their clinical significance should be evaluated in conjunction with cardiac functional status and overall circulatory conditions.

With respect to categorical clinical characteristics, no statistically significant associations were observed between age, sex, or family history of HCM and mortality risk in the present cohort. This finding is likely attributable to the marked etiological and phenotypic heterogeneity of pediatric HCM, together with the limited sample size and number of outcome events. Previous large-scale studies have suggested that prognosis in pediatric HCM is more strongly influenced by age at presentation, underlying etiology, and baseline cardiac functional status than by individual discrete clinical variables (22, 23). Accordingly, the absence of statistical significance for categorical variables in this study should be interpreted as reflecting limited statistical power rather than excluding their potential prognostic relevance in larger populations.

Female sex demonstrated only a borderline association with mortality (*p* = 0.085) in the present cohort. Prior studies have reported inconsistent findings regarding sex-related prognostic differences in pediatric HCM, particularly after accounting for genetic background and disease stage (38–41). Similarly, family history of HCM was not significantly associated with mortality, although previous evidence suggests that family history—especially of sudden cardiac death—may carry prognostic value in specific genetic contexts (42–45). In pediatric populations, however, the predictive impact of family history may be attenuated by a higher proportion of sporadic cases, *de novo* mutations, and age-dependent penetrance.

In addition, no significant associations were observed between routine electrocardiographic variables and mortality risk. This may reflect the use of binary ECG classifications without phenotype-specific stratification, as well as limited statistical power. These findings highlight the need for future studies employing more refined, phenotype-oriented electrocardiographic assessment in larger pediatric HCM cohorts.

### 4.1 Strengths, limitations, and future directions

This study has several strengths, including its real-world design, clearly defined cardiovascular-related mortality endpoint, relatively long follow-up duration, and the application of X-tile to derive outcome-oriented cut-off values for continuous variables. However, important limitations must be acknowledged. These include the retrospective single-center design, small sample size with a limited number of outcome events, which may affect the stability of hazard ratio estimates, reliance on univariate analyses, and etiological heterogeneity encompassing primary, secondary, and unclassified HCM. Given the limited number of outcome events, multivariable Cox regression analysis was not performed to avoid potential overfitting and unstable estimates. In addition, the absence of body surface area adjustment or Z-score standardization for echocardiographic parameters and the potential risk of overfitting inherent to data-driven cut-point selection should be considered. This limitation may be particularly relevant given the relatively small sample size of the present study. Future multicenter studies with standardized phenotyping, refined electrocardiographic stratification, etiological and genetic subclassification, and external validation of risk thresholds are needed to improve generalizability and clinical utility.

## Data Availability

The raw data supporting the conclusions of this article will be made available by the authors, without undue reservation.
